# Building a knowledge base to assist clinical decision-making using the Pediatric Research Database (PRD) and machine learning: a case study on pediatric asthma patients

**DOI:** 10.1186/1471-2105-15-S10-P17

**Published:** 2014-09-29

**Authors:** Naga Nagisetty, Eunice Y Huang, Grady Wade, Teeradache Viangteeravat

**Affiliations:** 1 Biomedical Informatics Core, Children’s Foundation Research Institute, Memphis, TN, 38103, USA; 2 Department of Pediatrics, University of Tennessee Health Science Center, Memphis, TN, 38163, USA

## Background

The Pediatric Research Database (PRD) is a clinically rich de-identified, standardized database designed around our institution's electronic medical record (EMR) system. We intend to expand the utilization of the PRD to assist physicians by providing historical patterns based on conversations among physicians, patients and research personnel. As an example of such implementation, we are currently using a prevalent, chronic respiratory disease, i.e., asthma.

Asthma is a very common disease in children. Early identification of patients at high risk of developing asthma can help provide them the best possible treatment. Identifying such patients from huge data sets (e.g., EMRs) is challenging and very time consuming. Using data mining techniques [1-3] to learn from past examples not only permits researchers to detect expected events, such as might be predicted by models, but also helps to discover unexpected patterns and relationships that may provide new insights.

## Materials and methods

For this preliminary study, we acquired de-identified data sets from the PRD for patient visits in 2012. The total number of observations included 92,175 encounters. We selected encounters with APR-DRG codes = 141 Asthma, 144 Respiratory signs & minor diagnoses, 131 Cystic fibrosis – pulmonary disease, and 132 BPD & chronic respiratory disease our initial datasets. The total number of encounters meeting the criteria was 8,895, including 7,011 distinct patient records.

## Results

Among all patients, 57.8% (4,052) were male, 11.7% (817) were white, and 81.1% (5,685) were black or African-American. Each contributing factor is analyzed to focus the algorithm used to suggest relevant information to the physicians. The rules are not built to predict outcomes, but to provide physicians with facts and relevant associations from existing records to confirm a therapeutic approach and suggest optimal treatment.

Current models in the PRD are restricted to a limited set of variables, such as demographics (age, weight, gender, body mass index, zip code), admission diagnoses, primary and secondary diagnoses, APR-DRGs, and imaging information. Ongoing development will lead to inclusion of medications, generic laboratory information, and publicly available data from the Food and Drug Administration Adverse Event Reporting System (FAERS) to augment information available to physicians.

## Conclusions

Access to a knowledge-based clinical support decision system at the point-of-care is the foundation of evidence-based health care. We believe this knowledge base provides physicians with an opportunity to review a history of similar cases and outcomes at the time of providing care, thereby assisting in decision-making.
